# Co-expression of a retrotransposon and re-targeted restriction factor impairs yeast growth

**DOI:** 10.17912/micropub.biology.001905

**Published:** 2025-11-03

**Authors:** Sean L Beckwith

**Affiliations:** 1 Department of Biology, Hope College, Holland, Michigan, United States

## Abstract

Retrotransposons and retroviruses shape genome evolution and can negatively impact genome function. *Saccharomyces cerevisiae* minimizes replication of Ty1 family retrotransposons using an inhibitory protein identical to the C-terminal domain of the transposon’s capsid protein. We previously elucidated the molecular basis of restriction and subfamily specific targeting and engineered a re-targeted restriction factor. Here, we report that co-expression of the Ty1' retrotransposon and a variant of its restriction factor mutated to re-target a related transposon subfamily impairs yeast growth.

**Figure 1. Drt2m-AVL impairs yeast growth when co-expressed with Ty1':**
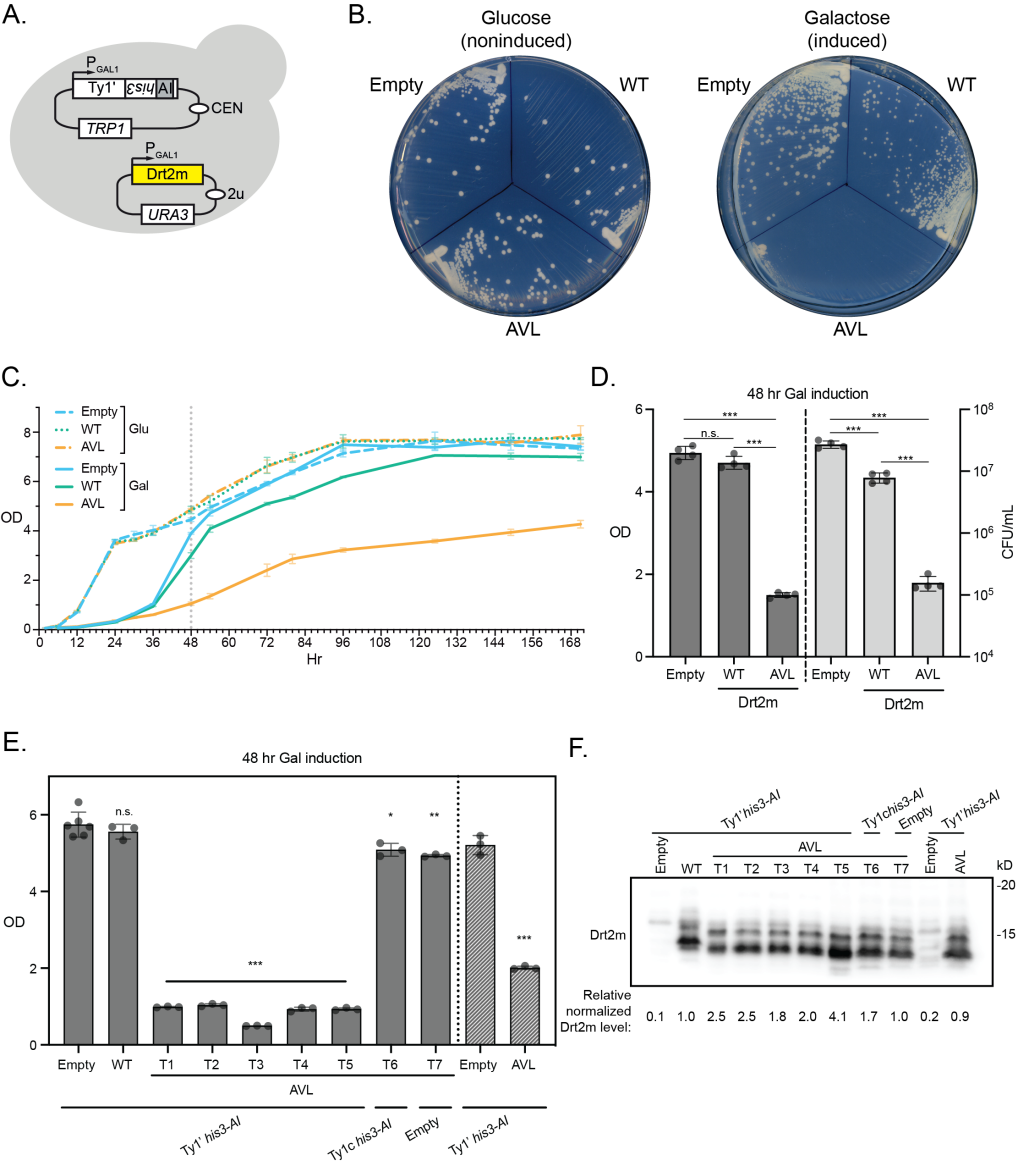
( **A** ) Schematic illustrating the two-plasmid yeast strain construction used. The *URA3* plasmid (yellow) contained either Drt2m (WT), Drt2m-AVL (AVL), or empty vector (Empty). ( **B** ) Representative images showing growth of single colonies on agar plates containing glucose (left) or galactose (right). ( **C** ) Growth curve over 7 days measured by OD _660_ in glucose (Glu) or galactose (Gal) media. ( **D** ) Comparison of cell growth after 48 hr of growth in galactose media measured either by OD _660_ or by calculating colony forming units (CFU) per mL of culture. ( **E** ) Cell density measured by OD _660_ after 48 hr of growth in galactose media of a panel of strain constructions (see main text for explanation of T1-T7). The lower labels indicate the Ty1 transposon present and the upper labels indicate the Drt2m allele present. Hatched bars on the right indicate a different yeast strain background. For panels C-E, error bars represent standard deviation; n.s. = not significant, *** *p* < 0.001, ** *p* < 0.01, * *p* < 0.05; see Extended Data for raw data and *t* -test comparisons. ( **F** ) Western blot of whole-cell extracts from the strains presented in (E) detecting hexahistidine-tagged Drt2m alleles. Total protein per lane was measured (see Extended Data) and used to normalize Drt2m protein levels, indicated below the blot. Molecular weight standards are indicated to the right.

## Description

Through reverse transcription, retrotransposons create a copy of themselves which is subsequently inserted into the host genome. Each transposition event, therefore, increases total copy number and can impact genome evolution. A variety of transposon control mechanisms have evolved to prevent excessive replication, including RNAi pathways, and SAMHD1, APOBEC, and MOV10 restriction factors (Goodier, 2016). The budding yeast *Saccharomyces cerevisiae* and the closely related species *S. paradoxus* lack these defense mechanisms, yet transposons comprise only a small fraction of the yeast genome (Garfinkel et al., 2016). *S. cerevisiae* contains several related groups of long-terminal repeat (LTR)-retrotransposons, the most abundant being Ty1 in many strains (Bleykasten-Grosshans et al., 2021; Curcio et al., 2015). The Ty1 genome encodes for Gag and Pol proteins. Ty1 Gag provides both capsid (CA) and nucleic acid chaperone functions. Ty1 Pol is a polyprotein that is proteolytically processed into protease, integrase, and reverse transcriptase enzymes, each of which is required for retrotransposition. Ty1 Gag assembles cytoplasmic virus-like particles (VLPs) where Ty1 protein maturation occurs and Ty1 mRNA is reverse transcribed (Curcio et al., 2015; Garfinkel et al., 1985). Ty1 Gag contains N- and C-terminal CA domains (NTD/CTD) that assemble into Ty1 VLPs (Cottee et al., 2021).

*S. cerevisiae* restricts uncontrolled retrotransposition of Ty1 subfamilies using distinct mechanisms: canonical Ty1 (Ty1c) is inhibited by a self-encoded restriction factor, p22/p18, whereas the closely related Ty1' is inhibited by an endogenized restriction factor, Drt2 (Cottee et al., 2021; Hannon-Hatfield et al., 2024; Saha et al., 2015). The minimal inhibitory fragment of both restriction factors (p18m and Drt2m) is a conserved CA C-terminal domain (CTD) which interacts with their respective CA via an extensive hydrophobic interface termed “Dimer-1”. We recently reported that p18m and Drt2m are highly specific to their subfamilies (Beckwith et al., 2025). There are only three amino acid residues found within Dimer-1 that diverge between the subfamilies but are highly conserved within each subfamily: A266, V270, and L312 (AVL) in Ty1c and p18 compared to V266, T270, and F312 (VTF) in Ty1' and Drt2. By mutating these three residues, we re-targeted each restriction factor to the opposite transposon.

Here, we demonstrate that the re-targeted restriction factor, Drt2m-AVL, causes a growth defect specifically when co-expressed with the Ty1' retrotransposon. We expressed the Ty1' retrotransposon, marked with the *his3-AI* retrotranscript indicator gene (which enables monitoring of mobility frequency), on a plasmid under the *GAL1* galactose-inducible promoter (Curcio & Garfinkel, 1991) ( **Figure 1A** ). Wildtype Drt2m or the re-targeted mutant Drt2m-AVL were tagged with a C-terminal hexahistidine tag and expressed alongside Ty1' *his3-AI* on a separate plasmid, also under the control of the *GAL1* promoter. Experiments were performed in a *S. paradoxus* yeast strain lacking any full-length Ty elements (Chen et al., 2022; Cottee et al., 2021; Saha et al., 2015).

We observed that co-expression of Drt2m-AVL and Ty1' drastically impaired cell growth compared to wildtype Drt2m or empty vector. We measured this growth defect in both plate and liquid assays and across different time points. On agar plates, cell growth is not impaired when the strains are grown on glucose media which represses gene expression from our constructs controlled by the *GAL1* promoter ( **Figure 1B** ). When expression is induced by growing cells on galactose plates, wildtype Drt2m expression causes a slight growth reduction as seen by colony size. However, Drt2m-AVL expression results in extremely reduced colony size. We further measured liquid growth of these three strains in non-inducing (glucose) and inducing (galactose) media over more than 1 week (171 hours). Yeast cells grow more quickly in glucose, their optimal carbon source, than in galactose. In glucose, growth of the three strains is not dramatically different ( **Figure 1C** , see **Extended Data** for *t* -test comparisons). However, when Drt2m-AVL is expressed on galactose, growth of the culture is dramatically slowed. Expression of wildtype Drt2m slightly reduces culture growth but the culture still saturates and growth plateaus by the end of the data collection period. The strain expressing Drt2m-AVL never approached a cell density close to empty vector or non-induced cultures. We selected the 48 hr time point to further examine growth in liquid media as Drt2m-AVL displayed a marked growth defect. We validated optical density (OD) as an accurate, rapid measure of cell density of the culture after 48 hr in galactose media. Drt2m-AVL expressing cultures had significantly lower OD than wildtype Drt2m and empty vector strains and had one to two orders of magnitude fewer colony forming units (CFU) per mL of culture ( **Figure 1D** ).

We next tested the growth defect of Drt2m-AVL co-expressed with Ty1' in several different strain constructions by measuring OD after 48 hr of growth in galactose media. The growth defect was observed in strains generated through four independent transformations (T1-T4) and a strain generated by transforming in the Drt2m-AVL and Ty1' *his3-AI* plasmids in the opposite order (T5). Co-expression of Drt2m-AVL with Ty1c *his3-AI* (T6) or with an empty vector (T7) did not dramatically impair growth ( **Figure 1E** ). Furthermore, the growth defect was reproducible in a distinct strain background of Ty-less *S. paradoxus* ( **Figure 1E** , hatched bars on right). Finally, we assessed Drt2m and Drt2m-AVL protein expression by western blot. All strains expressed protein well with some variability in protein level (see **Extended Data** for quantification of total protein levels). Protein expression does not appear to explain the striking growth defect especially when Drt2m-AVL and Ty1' are co-expressed.

In summary, we have shown a marked growth defect in yeast co-expressing Drt2m-AVL and Ty1' both on agar plates and liquid media. This defect is not an artifact of plasmid construction as it is only observed in galactose media that induces gene expression. Wildtype Drt2m expression causes a slight reduction in growth seen qualitatively by colony size on agar plates but inconsistently in liquid media measured by OD _660_ at 48 hr. The dramatic growth impairment caused by Drt2m-AVL expression is consistently observed on solid and liquid media and is reproducible in several strain constructions and in two *S. paradoxus* backgrounds. Differences in protein expression do not explain the impaired cell growth. The severe growth defect is only observed when Drt2m-AVL and Ty1' are both expressed, not when Ty1' is expressed with wildtype Drt2m nor when Drt2m-AVL is expressed with Ty1c or in the absence of any transposon. The mechanism behind this growth defect remains to be determined but may involve cellular products that modulate the process of Ty1 retrotransposition (Curcio et al., 2015).

## Methods

*Yeast strains, plasmids, and media*

Yeast strains and plasmids used in this study are included under **Reagents** . All yeast strains are Ty-less *Saccharomyces paradoxus* . SLBY470 and SLBY471 were generated by transforming the indicated plasmids into strain DG2204 (Beckwith et al., 2023). Yeast was grown in synthetic complete (SC) media lacking uracil (-U) and tryptophan (-T) and containing the indicated sugar at 2% w/v. For induction in liquid media, starter cultures were grown overnight at 30 °C in synthetic media containing raffinose, diluted 1:20 into media containing either galactose or glucose, and grown at 22 °C. Optical density (OD) readings were measured at 660 nm; readings greater than 1.0 were diluted 1:10 in water and the diluted value was multiplied by 10. Colony forming units (CFU) per mL of culture were calculated by diluting culture in sterile water, plating on SC-U-T glucose plates, and counting colonies after four days of growth at 30 °C (see **Extended Data** for dilution factors and volumes). All graphs show data from at least three replicates and colony growth on plates are representative images of three replicates.

*Protein extraction and western blotting*

Western blotting was performed on total protein extracted from galactose-induced yeast by trichloroacetic acid (TCA) precipitation. Cells were broken by vortexing in the presence of glass beads in 20% TCA for 5 min, cooling on ice for 3 min, and vortexing again for 5 min. Glass beads were washed in 5% TCA; the liquid was transferred to a new tube and centrifuged at 9,391 x g for 10 min. The pellet was resuspended in 150 μL of 4x Laemmli Sample Buffer (Bio-Rad cat. no. 1610747) containing 2-mercaptoethanol and boiled for 5 min. Proteins were separated on AnyKD Stain-Free Protein Gels and visualized according to supplier specifications (Bio-Rad cat. no. 4568126). PVDF membranes were immunoblotted with monoclonal mouse hexa-histidine antibody clone HIS.H8 (ThermoFisher cat. no. MA1-21315) diluted 1:3000 in 2.5% milk-TBST and then peroxidase-conjugated anti-mouse IgG (Cytiva cat. no. NA931) diluted 1:5,000 in TBST. Immune complexes were detected with WesternBright enhanced chemiluminescence (ECL) detection reagent (Advansta cat. no. K-12049-D50). Imaging was performed using a ChemiDoc MP (Bio-Rad). Total protein normalization and band quantification was done using Image Lab software (Bio-Rad). Precision Plus Kaleidoscope protein standards (Bio-Rad cat. no. 1610395) were used to estimate molecular weights. The western blot shown is representative of three replicates.

## Reagents

**Table:** 

Yeast strains used in this study.							
Strain	Alias	Label	Genotype	Plasmids	*TRP1/CEN* plasmid:	*URA3/2μ* plasmid:	Source
SLBY431	DG4576	Empty	^a^	pBDG1697, pBDG1293	pGTy1' *his3-AI*	empty pYES2	Beckwith et al., 2025
SLBY435	DG4578	WT	SLBY431	pBDG1697, pBDG1758	pGTy1' *his3-AI*	Drt2m	Beckwith et al., 2025
SLBY433		T1	SLBY431	pBDG1697, pBDG1818	pGTy1' *his3-AI*	Drt2m-AVL	This study
SLBY446	DG4580	AVL (T2)	SLBY431	pBDG1697, pBDG1818	pGTy1' *his3-AI*	Drt2m-AVL	Beckwith et al., 2025
SLBY452		T3	SLBY431	pBDG1697, pBDG1818	pGTy1' *his3-AI*	Drt2m-AVL	This study
SLBY453		T4	SLBY431	pBDG1697, pBDG1818	pGTy1' *his3-AI*	Drt2m-AVL	This study
SLBY459		T5	SLBY431	pBDG1818, pBDG1697	pGTy1' *his3-AI*	Drt2m-AVL	This study
SLBY434	DG4575	T6	SLBY431	pBDG1534, pBDG1818	pGTy1c *his3-AI*	Drt2m-AVL	Beckwith et al., 2025
SLBY458		T7	SLBY431	pBDG1818, pBDG637	empty pRS414	Drt2m-AVL	This study
SLBY470		Empty	^b^	pBDG1697, pBDG1293	pGTy1' *his3-AI*	empty pYES2	This study
SLBY471		AVL	SLBY470	pBDG1697, pBDG1818	pGTy1' *his3-AI*	Drt2m-AVL	This study
^a^ Saccharomyces paradoxus MAT⍺ gal3 his3-Δ200hisG trp1-1* ura3 Ty-less							
^b^ Saccharomyces paradoxus MATa gal3 his3-Δ200hisG trp1-1* ura3 Ty-less							

**Table:** 

Yeast plasmids used in this study.			
Plasmid	Description	Markers	Source
pBDG1293	pGAL-Yes2	*URA3/2μ*	ThermoFisher cat. no. V82520
pBDG1758	pBDG1293-Drt2m-6xHis	*URA3/2μ*	Hannon-Hatfield et al., 2024
pBDG1818	pBDG1758-Drt2m-V266A/T270V/F312L	*URA3/2μ*	Beckwith et al., 2025
pBDG1697	pGTy1'his3-AI	*TRP1/CEN*	Hannon-Hatfield et al., 2024
pBDG1534	pGTy1chis3-AI	*TRP1/CEN*	Saha et al., 2015
pBDG637	Empty vector pRS414	*TRP1/CEN*	Brachmann et al., 1998

**Table:** 

Antibodies used in this study.			
Antibody	Species	Type	Source
anti 6x-His tag	Mouse	Monoclonal (clone HIS.H8)	ThermoFisher cat. no. MA1-21315
anti mouse IgG (HRP-conjugated)	Sheep	Polyclonal	Cytiva cat. no. NA931

## Data Availability

Description: Underlying data of charts and tables.. Resource Type: Dataset. DOI: https://doi.org/10.22002/nzrkb-9k850
